# Consumer willingness-to-pay for blockchain-based QR code traceability of leafy greens

**DOI:** 10.1371/journal.pone.0331614

**Published:** 2025-10-08

**Authors:** Alba J. Collart, Matthew G. Interis, Elizabeth Canales, Ajita Giri

**Affiliations:** 1 Department of Agricultural Sciences, Clemson University, Clemson, South Carolina, United States of America; 2 College of Agriculture, Forestry and Life Sciences, Clemson University, Clemson, South Carolina, United States of America; 3 Department of Agricultural Economics, Mississippi State University, Starkville, Mississippi, United States of America; Beijing Technology and Business University, CHINA

## Abstract

There is continued interest in the modernization of food traceability systems because of increased consumer consciousness of food production, processing, and transportation and the desire of the food industry to identify and contain sources of foodborne illness outbreaks and improve their reputations to consumers. Blockchain-based traceability systems promise faster access to more decentralized, tamper-proof records of the movement of a food product and its ingredients through the supply chain. Using data from two discrete choice experiment surveys, we estimate U.S. consumer marginal willingness to pay for access to blockchain-based traceability information via QR codes and for more specific sub-region provenance labeling placed on the packaging of two economically important and outbreak-prone leafy greens: romaine lettuce and spinach. After conducting sensitivity testing using a variety of specifications, we find that unrestricted random parameter logit models allowing for correlation across random parameters provide the best model fit. Simulations of willingness to pay distributions indicate a median marginal willingness to pay of about $1.45 for access to traceability information over no access and an additional $0.33-$0.38 if the information is blockchain verified. We also find that voluntary sub-region provenance labeling may result in consumers discounting imported relative to domestically produced leafy greens. If domestically produced, consumers are not willing to pay more to know the region within a state where the product was grown.

## Introduction

The recurring linkage between high-profile foodborne illness outbreaks and the consumption of leafy greens and consumers’ increasing attention to field-to-fork traceability have made the food industry and governments worldwide prioritize the improvement of traceability systems in the produce sector (see, e.g., [1,2]). In a June 2018 *E. coli.* multi-state outbreak in romaine lettuce in the United States, it took four months to identify the source farms of the outbreak, a wide variety of leafy greens (not just romaine) were discarded from shelves and restaurants around the nation, and 101 people either died or were hospitalized because of the outbreak. In November 2018, the U.S. Centers for Disease Control and Prevention (CDC) and the U.S. Food and Drug Administration (FDA) again warned consumers and food service operators against romaine, regardless of its growing region, after another *E.coli* romaine outbreak that spread across 16 states and resulted in 25 hospitalizations. The economic loss from foodborne illness outbreaks can be devastating, and even demand for non-contaminated products can be negatively impacted [3]. Other critical reasons for developing improved traceability systems include ensuring the integrity of programs certifying credence attributes, thereby protecting consumer trust and deterring food fraud. For instance, the U.S. Agricultural Marketing Service (USDA-AMS) has proposed improving farm-to-market traceability to increase the integrity and functionality of USDA regulations related to organic production [4].

Recent legislation is already putting growing pressure on the food industry to improve traceability. In 2020, the U.S. Food & Drug Administration (FDA) outlined a New Era of Smarter Food Safety, leveraging technology to achieve a safer and more digital, traceable food system, and in 2022, it published the Food Traceability Final Rule (FTL) to implement the Food Safety Modernization Act’s Rule 204(d) with a compliance date of January 2026. The FTL requires any U.S. or foreign company that manufactures, processes, packs, or holds specific high-risk foods, including leafy greens, to maintain digital traceability records for their products and generally respond to FDA information requests within 24 hours [5]. Consistent with a focus on tech-enabled traceability, some food industries are testing and implementing emerging digital traceability systems based on blockchain technology, including systems such as the IBM Food Trust in the United States and Europe and FreshChain in Australia. A highly publicized example is Walmart, which piloted the blockchain-based IMB Food Trust to trace the origin of mangos in 2016 and now requires leafy green suppliers to use [6]. Applications of blockchain technology aim to modernize food traceability by providing faster access to more decentralized, tamper-proof records of the movement of a food product and its ingredients through all steps in the supply chain. While there still are limitations and challenges for stakeholders to consider, adopting tech-enabled traceability systems might help the fresh produce industry with its need for better traceability systems [3,7].

Beyond public health reasons, adopting or improving traceability systems potentially yields several key benefits to the food industry. They can reduce liability in the event of a foodborne illness outbreak, as it is possible to more quickly identify the outbreak’s source and take appropriate measures to contain it. They can reduce costs incurred during an outbreak because the source can be more precisely identified, reducing the need, for example, to halt production or distribution or to dispose of product elsewhere in areas otherwise unaffected [8]. Additionally, there are potential benefits on the consumer demand side if consumers value improved traceability, the improved traceability is marketed to consumers accordingly, and market changes result in greater profits.

On the producer side, Stuller and Rickard [9] surveyed melon producers in California to identify the perceived benefits of food tracing. These benefits included improving production and distribution efficiencies and the firm’s reputation, mitigating litigation concerns, product differentiation, and adding value to the consumer. On the consumer side, the literature on the value of traceability information is vast (see [10] for a recent meta-analysis). Dickinson and Bailey [11] find that U.S. consumers are willing to pay for a traceability, transparency, and assurance system in meat, while Liu et al. [12] find that Chinese consumers value many kinds of traceability information for vegetable, dairy, and pork products including picking or slaughtering date, pesticide and fertilizer use, and information related to the producer, the packaging, and the transportation of the goods. However, Hobbs [13] warns that consumers may value traceability with quality assurances concerning specific credence attributes more than traceability by itself. Moreover, food risk perceptions may partly explain consumer willingness to pay for reliable tracing information of quality attributes [14]. A relevant concern in the produce sector is the risk of foodborne illnesses, which consumers are willing to pay a premium to reduce [15].

More recent studies have specifically examined the role of blockchain technology in verifying food production attributes. Sander et al. [16] find that the use of blockchain technology for food traceability significantly positively influences consumer purchasing decisions for meats. Li et al. [17] discuss the role of blockchain technology in tracing and verifying organic milk and wine production in China and generally find that its use increases consumer value. Contini et al. [18] examine the use of blockchain for tracing organic and certification attributes of craft beer in Italy and similarly find that it increased consumer demand. Lakkakula, Bullock and Wilson [19] find that using blockchain to verify soybean protein quality in the United States can result in “substantial” premiums. And in a U.S. study of beef, Shew et al. [20] find that while consumers are willing to pay a premium for blockchain-based verification of product source and other attributes, other verification systems such as those through the USDA, might provide even greater value. In a meta-analysis of consumers’ value for food traceability, Tran et al. [10] find that communicating the specific technology used to implement food traceability – including blockchain technology – can increase the price consumers are willing to pay for the product by up to 9%.

The purpose of our study is to examine U.S. consumer preferences for access to blockchain-based traceability information provided through QR codes and for more specific sub-region provenance labeling on packages of leafy greens, as manifested in their marginal willingness to pay. In particular, we examine these issues in the context of two economically important and disease-outbreak-prone leafy greens that are affected by recent industry implementations of blockchain and provenance labeling guidelines: spinach and romaine lettuce. Industry-issued and FDA-encouraged voluntary guidelines to label provenance (i.e., growing region and sub-region) remain in effect for romaine lettuce after two foodborne illness outbreaks impacted the industry in 2018, and similar guidelines may extend to other leafy greens in the future. Moreover, most U.S. consumers have the ability today to scan QR codes when out shopping or at home using their mobile devices. While QR codes could be used to provide information on food traceability to concerned consumers without the use of blockchain technology (e.g., via a website), blockchain technology may yet affect consumer trust in the information provided. Indeed, companies such as Carrefour and Nestlé currently provide blockchain-based traceability information through QR codes on food packaging with the marketing aim of food transparency for consumers in Europe [21]. While there are examples of blockchain-based food tracing in the United States, none so far are customer-facing, via the use of a QR code on the packaging or otherwise.

Our study makes three main contributions to the literature. First, we provide one of the first studies to examine the potential benefits of digital and blockchain-based food traceability as valued by food consumers, specifically for leafy greens. Therefore, we explore an important nexus of the literature on food risk from leafy greens and on blockchain-based traceability that has thus far been applied mainly to other categories of food products. Continued efforts to increase our understanding of the consumer side of traceability and the means of implementing it are critical for making decisions about both implementing and marketing food traceability efforts. Second, we examine consumer preferences for voluntary sub-region provenance labeling (i.e., where, more specifically, within the country or state of origin the product was grown) guidelines in domestic and imported romaine and spinach. Sub-region provenance labeling may be of interest to consumers who are concerned about their food’s origin and could help leafy green producers identify the contaminated product and reduce food loss and financial losses [22]. Third, we compare model fit and inferences for a comprehensive set—relative to existing studies in the literature—of discrete choice models. In particular, we examine models with correlated random parameters and error components in the utility specification, which have long been recommended [23,24], but are less applied in practice. We add evidence to the literature finding that the use of both correlated random parameters and error components increases model fit relative to more commonly used models and use the results of this model for each leafy green to simulate marginal willingness to pay (MWTP) values.

## Methodology

### Survey and discrete choice experiment

We designed two online surveys, each containing a discrete choice experiment (DCE), among primary grocery shoppers across the United States. The surveys were administered by Qualtrics between Feb 1—Mar 16, 2021, after approval from the university’s Institutional Review Board (protocol 20-410) and included a written consent form. The screening criteria for each survey included respondents who were primary grocery shoppers for their households, resided in the United States, were 18 years of age or older, owned at least one device with QR-code reading capabilities (e.g., a smartphone or tablet), and stated a written commitment to providing quality answers. In addition, we ensured specific quotas related to gender, age, and race, which we discuss in the results section. Depending on the DCE, respondents had to have purchased any packaged romaine lettuce or spinach sometime in the past month. Insights on consumer preferences for the use of blockchain technology in the tracking of romaine lettuce and spinach are of interest to industry policymakers and other researchers for a few reasons. These two leafy greens are widely consumed at home and away from home, are healthful yet commonly known to be prone to foodborne illness outbreaks and were among the first applications of blockchain-based farm-to-store traceability by U.S. mass retailers such as Walmart. In addition, respondents’ familiarity with these products facilitates decision-making and the evaluation of product attributes in the context of DCEs.

We conducted a pilot launch (50 completed responses for each product) before the final launch of each survey. To enhance data quality, we added a speeding check, automatically removing respondents who took less than one-half the median time to complete the survey, as measured during the pilot launch. This check helps increase confidence that participants respond thoughtfully and do not straight-line or click rapidly through the survey. We also included an attention check question or filter in the middle of the choice experiment [25]; after a participant completed half of the choice sets, they saw a Likert-scale attention check question that asked them to select “somewhat disagree” if they were 18 years of age or older. If they failed to choose “somewhat disagree,” they were screened out. Moreover, we deleted as protest responses all respondents who chose “I would not purchase any of these options” in all choice sets as their preferred alternative [26]. In the final launch of the survey, we received 500 completed responses for each product that also passed the speeding and attention checks (as contracted with the survey company). From the romaine survey, 1142 responses did not pass one of these two checks and were therefore excluded, with 914 responses not passing one of these two checks in the spinach survey. We removed four protest responses from the choice experiment dataset related to romaine lettuce and two from the spinach dataset. After enforcing all screening, quota, and data quality criteria, we had a usable sample size of 994 respondents in total, with 496 respondents from the final launch of the romaine survey and 498 respondents from the launch of the spinach survey.

Each survey started with an introduction describing instructions, the contact information of the researchers, and the process of consent to the research procedures. After a brief introduction, we asked respondents six true or false questions about blockchain technology to elicit their prior knowledge. Respondents were then provided paragraphs with information on blockchain, the implications to consumers of its use for traceability in the food industry, and how consumers would access the information using QR codes (S1 and S2 Files in supporting information) and quizzed again. Evaluating their knowledge before and after receiving information allows us to ensure their understanding of blockchain technology and its use for food tracing before starting the DCEs. Each survey also included a cheap talk script to mitigate any hypothetical bias in our DCEs [27]. A cheap talk script makes respondents aware of hypothetical bias and exhorts them to make their upcoming choices as if they were in a real-life setting (S3 File). Previous studies have shown the effectiveness of these scripts in reducing hypothetical bias [28–30]. After the cheap talk script, respondents learned the meaning of each attribute and attribute level and subsequently completed a series of choice questions.

Table 1 shows the choice experiment attributes and their levels related to organic certification, QR code traceability information, provenance labeling, and price. The USDA-certified organic label attribute indicated whether the product is certified organic by the U.S. Department of Agriculture or not. The QR code traceability information attribute referred to whether the package provided additional traceability information accessible through a blockchain-based QR code or a standard (not blockchain-based) QR code or whether there was no QR code. Importantly, the traceability information provided explicitly by the QR codes was described as information about the package’s journey from the farm to the store and, if relevant, a copy of the product’s organic certificate and a notice if there’s an active recall on that package. Digital traceability platforms such as IBM Food Trust in the United States and IDlocate in New Zealand, for example, offer the technology to provide such traceability information to consumers. Quality and nutrition perceptions, food safety, and environmental sustainability concerns regarding chemical runoff or the carbon footprint of longer supply chains may influence consumers’ demand for organic or traceable foods. Whether consumers are willing to pay premiums for these attributes may be of interest to industry stakeholders as pressure to improve traceability grows, and the sector is leading the way in conducting and implementing blockchain traceability systems. It may also interest government regulatory agencies such as the U.S. Food and Drug Administration (U.S. FDA) as they implement the Food Safety Modernization Act (FSMA) and their New Era of Smarter Food Safety initiative and consider technologies that could improve the traceability of leafy greens and other high-risk foods, and the USDA-AMS as they foresee future implementations of blockchain traceability systems to deter fraud and strengthen the National Organic Program [4].

**Table 1:** Discrete Choice Experiment (DCE) Attributes and Levels.

Attribute	Levels
USDA-certified organic label	No^a^
	Yes
QR code traceability information	No QR code^a^
	Standard QR code
	Blockchain-based QR code
Country of origin^b^	USA
	Mexico
Growing region information	CA sub-region known
	CA sub-region unknown^a^
	AZ sub-region known
	AZ sub-region unknown
	MX sub-region known
	MX sub-region unknown
Price (US$/3-count package)	$1.99, $2.49, $2.99, $3.49, $3.99, $4.49
Price (US$/1-lb. package)	If DCE with romaine lettuce hearts.
	$2.49, $2.99, $3.49, $3.99, $4.49, $4.99
	If DCE with baby spinach.

^a^Used as reference levels in experimental design.

^b^Attribute not included in experimental design as it can be inferred from the growing region information attribute.

In response to recent *E. coli* outbreaks, an ongoing U.S. FDA-industry initiative to improve the traceability of leafy greens is the voluntary labeling of detailed growing region (harvest location) and harvest date in romaine lettuce. We examine consumer preferences for knowing the more specific growing region within the country or state in which leafy greens are grown by including an attribute indicating whether the package had information on the growing sub-region in addition to the growing region. The growing region included California and Arizona, which account for most (90%) of the leafy greens produced (by weight) in the United States. It also included Mexico because the United States imports a small share of domestic consumption from Mexico [31]. For each region, the package could specify the sub-region (i.e., a more specific area within the states of California or Arizona or within Mexico) or not. The package had a map of the state or country delineating the sub-regions as classified by the U.S. Produce Marketing Association and the United Fresh Produce Association if it had additional information on the growing sub-region. The package had a map with an outline of the state or country if it had information on the growing region only. This experimental design allows us to separately measure the effects of growing region information and the QR code traceability attribute on consumer willingness to pay. Also, the country of origin on the packaging denoted the country where the product was grown. This attribute was not part of the experimental design as it can be inferred from the growing region information attribute (e.g., if the growing region was in California, the corresponding country of origin was the United States). The price of romaine lettuce refers to the price of a 3-count package of romaine lettuce hearts in U.S. dollars, and the price of spinach refers to a 1-lb. package of baby spinach in U.S. dollars. Price levels were balanced and varied in $0.50 increments from $1.99 to $4.49 for romaine lettuce and from $2.49 to $4.99 for spinach. To guide the selection of our price levels, we used the national-level market prices reported in the National Retail Report for Specialty Crops published by the USDA-Agricultural Marketing Service.

We generated the experimental designs in Ngene 1.2.1 [32] using a Bayesian D-efficient design approach (minimizing D-error). Efficient designs can lead to significantly lower standard errors but rely on accurate prior parameter estimates, which can be hard to identify beforehand [33]. Instead of assuming fixed prior parameters, Bayesian D-efficient designs consider the priors to be random parameters [32]. A Bayesian design approach assumes a prior distribution of probable parameter values and optimizes the design over that distribution. The Bayesian priors account for uncertainty about the prior parameter values and lead to a more robust efficient design. We generated Bayesian D-efficient designs assuming the conditional logit model (CL), which can also be efficient for estimating the random parameter logit (RPL) model [26,33].

In the first stage, we determined the prior estimates for the pilot launch of the romaine DCE using researchers’ expectations and a naïve approach [34]. In the second stage, we used Random Parameter Logit (RPL) modeling of responses from the pilot launch of the romaine DCE to update the Bayesian priors and generate the design used in the final launch of the romaine DCE. In the third stage, with more information about consumer preferences for leafy greens and prior parameter values, we used RPL modeling of responses from the final launch of the romaine DCE to determine the prior estimates for the pilot launch of the spinach DCE. This approach of using RPL modeling of the final launch of the romaine DCE instead of starting with a naïve approach to determine the prior estimates for the pilot launch of the spinach DCE generated a more efficient design. In the fourth stage, we used RPL modeling of responses from the pilot launch of the spinach DCE to update the Bayesian priors and generate the design used in the final launch of the spinach DCE. The D-error measures decreased with each subsequent stage as we updated the priors using additional information.

At each stage, the design we generated was a fractional factorial design consisting of 24 choice sets blocked into three blocks of eight choice sets, such that each respondent saw eight choice sets. Each choice set was comprised of four options. Options 1–3 offered leafy green products with certain levels of attributes, and Option 4 was the option to buy nothing, included to resemble a real-life setting in which shoppers may evaluate the available products but decide not to make a purchase. We randomly assigned each respondent to one of the three blocks and randomly presented the choice sets within each block to respondents. The choice questions used images and clear labeling of all the attribute information to aid the respondents’ understanding. S6 and S7 Files provide example choice sets as seen by participants in the romaine lettuce and spinach DCEs, respectively (note that respondents saw everything in these images, including the text and dashed boxes highlighting the attribute information on the packaging). Given the number of final respondents and the number of choice sets seen by all respondents, the total number of observed choices was 3,968 (496 respondents × 8 choice sets) in the romaine DCE and 3,984 (498 respondents × 8 choice sets) in the spinach DCE. In addition to the choice questions, each survey also collected demographic and behavioral information such as age, gender, race, education, employment status, income, marital status, household size, the number of children in the household, use of QR codes, and purchasing behavior.

### Conceptual framework and econometric model

The conceptual framework used in this study is random utility theory. Rational individual *i*, facing choice set *t*, will choose among *J* mutually exclusive options, each of which yields utility, *U*_*ijt*_:


$$U_{ijt}=V\left(X_{ijt}\right)+e_{ijt}\;j=1,\ldots l,\ldots J$$ (1)

where *V* is the deterministic portion of utility, ***X*** is a vector of attribute levels defining the options, and *e* is the random portion of utility. Although we assume that consumers are rational and have complete information about their preferences, the analyst has incomplete information about these preferences and *e* captures this uncertainty. Different assumptions about the distribution of *e* result in various discrete choice models [35]. Within a choice set *t*, the probability that individual *i* chooses alternative *l* can be described as:


$$P_{il}=\text{Pr}\left(U_{il}>U_{ij}\right)\;\forall j\neq l$$ (2)

$$P_{il}=\text{Pr}\left(V_{il}-V_{ij}>e_{sij}-e_{il}\right)\;\forall j\neq l$$ (3)

Because each individual saw eight choice sets with four options per choice set, the appropriate model would thus be analogous to a pseudo-panel data model in which the cross-sectional component is given by the individual, *i*, and the time-series component is given by the choice situation, *t.*

Conditional logit (CL) models and random parameters logit (RPL) models are commonly used to estimate the parameters for these types of discrete choice specifications. An RPL model extends the conditional logit model by relaxing the assumption of independence of irrelevant alternatives and accounting for consumer heterogeneity by allowing coefficients to vary across the population [24]. However, an RPL model introduces other complexities when deriving the distribution of MWTP for an attribute. For computational convenience, many studies estimating RPL models routinely assume a fixed price parameter to ensure that the resulting distribution of MWTP has the same distribution as the attribute’s coefficient, scaled by the price coefficient [36]. This assumption can be hard to maintain as it implies that all consumers have the same marginal utility of income [37,38] and has been discouraged [39].

One way to allow for preference heterogeneity in the price parameter is to assume it follows a lognormal distribution. Unlike other distributions that span zero, the lognormal can ensure finite moments (i.e., mean, variance) for the derived distribution of MWTP [40], it can be used to ensure a theoretically consistent negative influence of price on utility, and if random parameters are assumed to be correlated, it can facilitate drawing from a multivariate distribution because it is a transformation of a normal distribution [41]. Since a commonly discussed disadvantage of the lognormal is the possibility of a long right-hand tail resulting in large mean MWTP values, the median MWTP has been reported in the literature and can provide plausible marginal MWTP values [39,40,42,43].

The high flexibility of the RPL allows for the modeling of correlation across parameters that are common across alternatives in each choice situation [41]. Deriving MWTP based on RPL models with independent random parameters might be the state of practice when modeling food choice behavior and conducting environmental valuations, but recent studies show that estimating unrestricted RPL models that allow for correlation across random parameters can improve model fit [23,24]. These studies encourage applied researchers to include these specifications if enough data are available to warrant identification and convergence [37,39,44]. Including correlations in the random coefficients allows us to account for a relationship between people’s preferences for one variable and another [24]. In our study, we might expect that if one person prefers a QR code providing access to blockchain-based over standard traceability information, they may also prefer a QR code providing access to standard traceability information over no QR code. Hole and Kolstad [37] find that allowing for correlation across random parameters and for preference heterogeneity in the price parameter affects model fit more than whether the model is specified in preference space or willingness-to-pay space. Besides correlation across random parameters, another issue considered when modeling food choice behavior is whether to allow for correlation across the utilities of the buying options by including an error component (EC) [45–47].

In S4 File, we conduct sensitivity analysis to our model specification across a conditional logit specification and several random parameters logit specifications. Here in the main body of the paper, we focus on our preferred model with the best model fit: a random parameters logit (RPL) model in which the price parameter is specified to follow a lognormal distribution in the population, other attribute parameters are specified to follow a normal distribution in the population, the random parameters are allowed to be correlated, and the utilities of the alternatives in which a product is purchased are allowed to be correlated through the inclusion of an error component. The utility that respondent *i* gets from choosing alternative *j* within choice set *t* can generally be specified as:


$$\begin{array}{c} U_{ijt}=\beta_{ASC}{NoBuy}_{ijt}+\beta_{p}{Price}_{ijt}+\beta_{1,i}{Organic}_{ijt}+\beta_{2,i}{BlockchainQR}_{ijt}\\ \;\;+\;\beta_{3,i}{NoQR}_{ijt}+\beta_{4,i}{CA\_Known}_{ijt}+\beta_{5,i}{AZ\_Known}_{ijt}+\beta_{6,i}{AZ\_Unknown}_{ijt}\\ \;\;+\;\beta_{7,i}{MX\_Known}_{ijt}+\beta_{8,i}{MX\_Unknown}_{ijt}+\eta_{it}+\;e_{ijt} \end{array}$$ (4)

where *j* identifies the four available options per choice set, including the no-buying option. NoBuy is an alternative-specific dummy variable equal to 1 for the no-buy option and 0 for all other options in the choice set, $\beta_{ASC}$ is an alternative-specific constant associated with the no-buy option, Price is a continuous variable corresponding to the price of a 3-count package of romaine lettuce hearts or a 1-lb. package of spinach, $\beta_{p}$ is the price parameter, the rest of the variables are dummy coded variables identifying the other attributes levels considered in the experimental design, and $\beta_{1}-\beta_{8}$ are taste parameters for these variables. Also, $\eta_{it}$ represents the error component and $e_{ijt}$ represents the usual unobserved random error term. Again, the error component captures the correlation across the utilities of the buying options and is shared by their utility specifications, but not by the utility of the no-buy option. We use NLOGIT 6 and use 2,000 Halton draws to estimate the model parameters.

### Marginal Willingness-to-Pay

Marginal willingness to pay (MWTP) is the change in willingness to pay (WTP) when a single, non-price attribute level of the product changes. That is, the difference between the WTP for a specific product and the WTP for that product when a single non-price attribute changes, *ceteris paribus*. The general calculation of MWTP is:


$$MWTP=-\frac{\beta_{k}}{\beta_{p}}$$ (5)

where $\beta_{k}$ is the estimated non-price attribute parameter, k=1,…,8, and $\beta_{p}$ is the estimated price parameter.

Using the estimated parameters and variance-covariance matrix of the estimated model, we simulate the MWTP distributions to incorporate the sampling variation in the point parameter estimates and the variation in preferences within the population as captured by the random parameters. This method is preferred to using only point estimates of the population mean to calculate MWTP because simulating the population MWTP distributions uses all the information in the population distribution rather than just the mean [41]. Also, even if some imposed assumptions on the random parameters improve model fit (e.g., assuming a log-normally distributed price parameter, allowing correlated random parameters), this method can quickly provide a good indication of whether the MWTP values are realistic [39]. We use the Krinsky and Robb [48,49] approach to simulate MWTP distributions, and account for any correlation of random parameters when conducting the draws (see [41]). Simulation results are based on 10,000 pseudo-random draws from the sampling distribution and 500 Halton draws from each random parameter distribution.

## Results and discussion

### Sample characteristics

Table 2 reports the summary statistics of various demographic variables in our samples and compare them with the U.S. population based on U.S. Census Bureau categorizations. We also show tests for the equality of a variable’s mean in each sample with the variable’s mean in the U.S. population. All respondents were the primary grocery shoppers for their households. Because we set specific quota constraints for gender and the 65 + age group, the samples represent the U.S. population in terms of these variables. The quotas for each sample were 55% female and 45% male respondents, and a maximum of 16% of respondents aged 65 years or older. In addition, the romaine sample represents the U.S. population reasonably well in terms of Black or African American identity, income distribution, as well as respondents aged 45–54. The spinach sample was comparable to the U.S. population regarding respondents aged 25–34. Relative to the U.S. population, respondents in both samples had higher educational attainment, larger households, and were mostly married (62–66%).

**Table 2:** Summary Statistics and Variable Definitions.

Variable	Definition	Romaine Sample	Spinach Sample (means)	U.S. Pop.^a^
Age 18–24	1 if age is 18–24 years; 0 otherwise	0.04***	0.04***	0.09
Age 25–34	1 if age is 25–34 years; 0 otherwise	0.19***	0.12	0.14
Age 35–44	1 if age is 35–44 years; 0 otherwise	0.29***	0.39***	0.13
Age 45–54	1 if age is 45–54 years; 0 otherwise	0.12	0.09***	0.13
Age 55–64	1 if age is 55–64 years; 0 otherwise	0.21***	0.21***	0.13
Age 65+	1 if 65 years of age or older; 0 otherwise	0.15	0.16	0.16
Female^b^	1 if female; 0 otherwise	0.53	0.55	0.55
Male^b^	1 if male; 0 otherwise	0.47	0.45	0.45
White race identity	1 if identifies as White only; 0 otherwise	0.80***	0.75	0.73
Black race identity	1 if identifies as Black or African American only; 0 otherwise	0.15	0.21***	0.13
Another or multiple race identity	1 if identifies as another race or multiple races; 0 otherwise	0.04***	0.04***	0.14
Associate^c^	1 if highest educational attainment is 2-year or associate degree; 0 otherwise	0.42***	0.39***	0.68
Bachelor’s^c^	1 if highest educational attainment is bachelor’s degree; 0 otherwise	0.30***	0.32***	0.20
Graduate^c^	1 if highest educational attainment is graduate school degree; 0 otherwise	0.28***	0.29***	0.12
Household size^d^	No. of persons per household	2.89***	2.92***	2.36
Children^d^	No. of <18-year-old-persons per household	0.87***	0.98***	0.53
Income $34,999 or less	1 if yearly household income before taxes is $34,999 or less; 0 otherwise	0.21***	0.19***	0.28
Income $35,000-$74,999	1 if yearly household income before taxes is $35,000 to $74,999; 0 otherwise	0.29	0.27*	0.30
Income $75,000-$99,999	1 if yearly household income before taxes is $75,000 to $99,999; 0 otherwise	0.15	0.16**	0.13
Income $100,000- $149,999	1 if yearly household income before taxes is $100,000 to $149,999; 0 otherwise	0.19**	0.26***	0.15
Income $150,000 or more	1 if yearly household income before taxes is $150,000 or more; 0 otherwise	0.16	0.11**	0.15
Part-time or full-time employed^e^	1 if employment status is part-time or full-time employed; 0 otherwise	0.66***	0.67***	0.60
Unemployed^e^	1 if employment status is unemployed; 0 otherwise	0.10***	0.10***	0.03
Stay at home parent or retired^e^	1 if employment status is stay at home parent or retired; 0 otherwise	0.24***	0.23***	0.37
Married	1 if married; 0 otherwise	0.62***	0.66***	0.48
Unmarried	1 if unmarried; 0 otherwise	0.38***	0.34***	0.52
South	1 if ZIP code is in the South; 0 otherwise	0.38	0.44**	0.38
West	1 if ZIP code is in the West; 0 otherwise	0.16***	0.13***	0.24
Midwest	1 if ZIP code is in the Midwest; 0 otherwise	0.17**	0.16***	0.21
Northeast	1 if ZIP code is in the Northeast; 0 otherwise	0.30***	0.27***	0.17
Number of respondents		496	498	

*Notes*: Single, double, and triple asterisks (*,**,***) indicate rejection of the null hypothesis at the 10%, 5%, and 1% significance levels, respectively, for a one-tailed t-test. Under the null, a variable’s mean in each group is equal to that variable’s mean in the U.S. population.

^a^*Source*: Categorizations based on 2015–2019 American Community Survey (ACS) 5-Year Estimates, U.S. Census Bureau population clock for 2019, and Food Industry Association.

^b^U.S. statistics for grocery shoppers ≥18 years old.

^c^U.S. statistics for population ≥ 25 years old.

^d^U.S. statistics calculated as variable’s total population divided by total housing units.

^e^U.S. statistics for population ≥ 16 years old. Employment categories in the ASC are: Employed civilian or armed forces in labor force, and not in labor force.

### Econometric models

S4 File contains our sensitivity analysis to the econometric model specification, but Table 3 shows the regression results from our preferred model with the best model fit. The standard deviation of the price parameter is highly significant for each product, and all non-price attribute parameters have statistically significant standard deviations as well, indicating that preferences are heterogeneous within the population. As for the estimates of the parameter means in the top half of the table, there is a strong consistency of which parameters are significant across the products. Notably, the mean of the price parameter is highly significant and negative (though recall it is restricted to be negative by its assumed lognormal distribution), providing strong evidence of price validity (a higher price decreasing utility, all else equal) in the data. The *No-buy* constant is negative and significant, indicating a tendency for respondents to choose to purchase a package of romaine lettuce, all else equal.

**Table 3:** Model Estimation Results.

	Spinach		Romaine	
	Parameter	(Clust. SE)	Parameter	(Clust. SE)
**Mean**				
Organic	1.169***	0.125	0.921***	0.106
Blockchain QR code	0.358***	0.095	0.293***	0.086
No QR code	−1.074***	0.105	−1.212***	0.097
CA sub-region known	0.114	0.140	0.058	0.107
AZ sub-region known	0.287**	0.135	0.187*	0.112
AZ sub-region unknown	0.027	0.135	0.069	0.118
MX sub-region known	−1.042***	0.165	−0.812***	0.154
MX sub-region unknown	−1.255***	0.166	−1.034***	0.166
Price	−0.605***	0.100	−0.355***	0.072
No-Buy	−10.616***	0.676	−6.346***	0.389
SD of Error Component (EC)	5.930***	0.639	2.793***	0.352
**SD of random parameters** ^**a**^				
Organic	1.512***	0.126	1.434***	0.206
Blockchain QR code	0.702***	0.096	0.765***	0.153
No QR code	1.025***	0.124	0.991***	0.230
CA sub-region known	0.703***	0.220	0.636**	0.312
AZ sub-region known	0.909***	0.184	0.847***	0.223
AZ sub-region unknown	0.881***	0.188	0.920***	0.271
MX sub-region known	1.663***	0.195	1.589**	0.740
MX sub-region unknown	1.721***	0.248	1.908**	0.794
Price	1.317***	0.099	0.714***	0.082
No. of observed choices (N)	3,984		3,968	
Log-Likelihood	−3,944.92		−4,178.05	
AIC	8,001.80		8,468.10	
BIC	8,354.10		8,820.10	

^a^All random parameters normally distributed, except for price which is lognormally (LN) distributed.

Estimated random parameters in all RPL models are based on 2,000 Halton draws.

***, **, * indicate statistical significance at the 1%, 5%, and 10% levels, respectively.

From the estimates of the attribute parameter means, we conclude that the average respondent prefers organic to non-organic lettuce, though the standard deviations on organic are large relative to their means, indicating considerable preference heterogeneity. For the QR code attributes, the average respondent prefers a QR code with traceability information verified with blockchain technology to the (omitted) standard QR code with non-blockchain-verified information. However, they prefer some QR code to none at all. Notably, there is preference heterogeneity in these QR code preferences. Regarding provenance labeling, recall that we use California with unspecified sub-regions as the omitted reference level. The parameters on the California and Arizona regions are generally not significant, suggesting no strong preference for sub-regions (in California) or regions (California vs. Arizona) of origin that are within the United States. However, the parameters on Mexico are negative and significant (again, with wide preference heterogeneity as indicated by the standard deviations when the parameters are specified to be random). Hence, while voluntary provenance labeling may not result in consumers strongly preferring leafy greens grown in one state over another, provenance labeling may result in U.S. consumers discounting imported products relative to domestically produced leafy greens.

### Marginal willingness-to-Pay

We use the estimation results for each type of leafy green to estimate marginal willingness to pay (MWTP) values. Because of the random parameters in the model, MWTP values will have a distribution within the population. Table 4, therefore, displays the estimated population mean MWTP, the estimated population median MWTP, the estimated proportion of the population whose MWTP is of the same sign as the median MWTP, and the 95% confidence intervals for each of these measures. The population mean and median MWTP values differ quite a bit for each attribute, indicating a skewed distribution of MWTP. The distributions seem more severely skewed for spinach than for romaine lettuce. For both products, the signs on the means and medians match what we would hypothesize *ex-ante* in many cases: on average, people prefer an organic product to a non-organic product, prefer any QR code to no QR code, prefer a QR code with blockchain-verified information to a QR code without blockchain-based verification, and, given the target U.S. population, prefer a product grown in California to one grown in Mexico. Given the skewness of the population distributions, we focus the remainder of our discussion on the population median MWTP values.

**Table 4:** Simulated Consumer Marginal Willingness-to-Pay for Leafy Green Attributes [95% CI].

Attribute	Population Mean MWTP [CI]		Population Median MWTP [CI]		% of Population with Sign(Median) [CI]	
*Romaine Lettuce (3-count package)*						
Organic	$0.67	[-0.05, 1.31]	$1.03	[0.74, 1.35]	74%	[67, 82]
Blockchain QR code	$0.47	[-0.12, 1.05]	$0.33	[0.13, 0.55]	65%	[55, 75]
No QR code	-$1.79	[-2.39, -1.2]	-$1.45	[-1.87, -1.1]	88%	[79, 98]
CA sub-region known	$0.58	[-0.16, 1.34]	$0.07	[-0.19, 0.33]	53%	[41, 64]
AZ sub-region known	$1.17	[0.5, 1.92]	$0.24	[-0.04, 0.55]	57%	[48, 67]
AZ sub-region unknown	$1.06	[0.36, 1.81]	$0.07	[-0.23, 0.39]	52%	[44, 60]
MX sub-region known	-$0.43	[-1.3, 0.55]	-$0.94	[-1.35, -0.57]	66%	[57, 77]
MX sub-region unknown	-$0.91	[-1.9, 0.22]	-$1.13	[-1.61, -0.69]	65%	[58, 72]
*Spinach (1-pound package)*						
Organic	$6.70	[3.66, 11.3]	$1.41	[1.06, 1.83]	78%	[71, 84]
Blockchain QR code	$1.36	[-0.05, 2.92]	$0.38	[0.16, 0.61]	69%	[59, 78]
No QR code	-$5.47	[-9.21, -3.09]	-$1.46	[-1.94, -1.07]	85%	[77, 93]
CA sub-region known	$0.78	[-1.37, 3.11]	$0.10	[-0.15, 0.39]	55%	[42, 71]
AZ sub-region known	-$1.19	[-4.24, 0.9]	$0.22	[-0.02, 0.50]	60%	[49, 71]
AZ sub-region unknown	-$1.35	[-4.18, 0.77]	-$0.02	[-0.3, 0.22]	50%	[40, 61]
MX sub-region known	-$3.75	[-7.5, -1.03]	-$1.14	[-1.61, -0.73]	71%	[63, 78]
MX sub-region unknown	-$4.33	[-7.91, -1.71]	-$1.39	[-1.89, -0.95]	72%	[65, 79]

*Note:* Results based on 10,000 pseudo-random draws from the sampling distribution and 500 Halton draws from each random parameter distribution.

In both the romaine lettuce and spinach samples, the 95% confidence intervals of the population median MWTP values lie entirely within the positive domain for the attributes of organic and the QR codes. This means we are reasonably confident that the median population member has a positive MWTP for an organic leafy green (relative to non-organic), for blockchain-verified QR code traceability information (relative to a standard QR code), and for standard QR code traceability information (relative to none). Simulation results indicate that consumers are willing to pay median price premiums of $1.03 (3-count romaine package) and $1.41 (1-lb. spinach package) for organic over non-organic leafy greens. They are also willing to pay median price premiums of $1.45 (romaine) and $1.46 (spinach) for a QR code providing access to standard traceability information over having no access to additional information and of $0.33 (romaine) and $0.38 (spinach) for a QR code giving access to blockchain-verified over standard traceability information.

In both samples, the 95% confidence intervals of the population median WTP values lie entirely within the negative domain for the attributes of the Mexico-grown products. Hence, we are reasonably confident that the median population member has a negative MWTP for a product grown in Mexico (relative to a product grown in California). If the growing region is unknown, consumers prefer domestically produced over leafy greens imported from Mexico. Consumers have median price discounts of $1.13 (romaine) and $1.39 (spinach) for leafy greens produced in Mexico relative to California. If the leafy greens are domestically produced, consumers have no strong preference for knowing over not knowing the detailed growing region (as evidenced by the confidence intervals for California and Arizona production attributes spanning zero).

The last column of Table 4 provides additional insight into the distribution of preferences within the population. For each attribute, it shows the estimated proportion of the population whose MWTP value has the same sign as the population median MWTP value. This measure is conceptually similar to the probability of direction measure commonly used in Bayesian econometrics for testing the presence of an effect [50]. By definition, this value will always lie between 50% and 100%, and the closer to 100% it is, the more confidently we might claim that “most” people in the population prefer or do not prefer a given attribute. Additionally, if its confidence interval is entirely above 50%, we are relatively confident that at least 50% of the population has a MWTP value with the same sign as the population median. For both products, the lower end of the confidence intervals for the organic, QR, and Mexico-grown attributes all lie above 50%. For both products, the attribute with the highest estimated proportion of the population having a MWTP of the same sign as the median is the “No QR code” attribute, which has a negative median MWTP. The next highest value for both products is for the organic attribute, followed by the Mexico-grown attributes. Looking more closely at MWTP for a QR code with blockchain-based information compared to a QR code with information that is not blockchain-based, we are relatively confident that most people prefer the former, but that majority could be as low as 55% (or as high as 75%) for romaine lettuce and as low as 59% (or as high as 78%) for spinach. With either product, there is likely still a fairly large proportion of the population who is unwilling to pay more for blockchain-based QR code information.

To examine heterogeneity of preferences for blockchain-based QR code information a bit further, we used the “;par” and “;WTP” commands in NLOGIT 6 to estimate an individual-level marginal willingness to pay value for this attribute for each person in the sample, and then regressed whether this MWTP value was positive (1 if strictly positive, 0 otherwise) on a set of respondent-specific demographic and behavioral variables in a logit model (We thank a reviewer for suggesting this additional analysis. S5 File contains the logit regression results). In the romaine sample, respondents were more likely to have a positive WTP for a blockchain-based QR code on the packaging if they were married, had a relatively lower income, lived in the northeast U.S. instead of the south or Midwest, or had concerns about food safety, as measured by them strongly or somewhat agreeing to the statement “I’m concerned with improving food safety of the U.S. food supply.” Notably, interest in food origin and how food systems work does not have a statistically significant effect. In the spinach sample, respondents were more likely to have a positive WTP if they had a relatively larger household, could correctly identify a QR code image (shown in the survey), or stated a relatively higher frequency of scanning food packaging QR codes at home. In contrast, the frequency of QR code scanning in store had no statistically significant effect. While the findings differ across products, the findings for each product could inform policy or marketing approaches designed to add value to the consumer experience through these kinds of traceability practices. For example, large-scale applications of blockchain-based traceability by mass retailers in the U.S. have primarily focused on how these systems could be used internally to improve food safety, manage recalls, and ensure compliance rather than offering consumers additional product information, such as food origin. Before scaling consumer access to blockchain-based QR code traceability, U.S. retailers could benefit from a deeper understanding of QR code user demographics and behaviors, particularly how their consumers interact with these technologies at home.

## Conclusions and future research

Overall, we find strong evidence that consumers value access to food traceability information for leafy greens and that if that information is verified using blockchain technology, the value of the information is higher. Depending on the costs of implementing or improving food traceability systems, including the potential use of blockchain for information verification, the value added to consumers has the potential to allow other supply chain participants to capture price premiums and yield increased profits. Of course, marketing would play an important role, as consumers would need to be aware of the availability and type of tracing information provided and the potential benefits of having access to it. While currently none of the applications of blockchain-based food tracing in the United States are customer-facing, companies such as Carrefour and Nestlé in Europe have communicated the availability of blockchain-based traceability information through QR codes using press releases and mass media articles with the aim of increasing consumer trust. We report estimates of mean and median consumer marginal willingness to pay to access traceability information. Future research can investigate consumers’ habits of using this kind of on-package traceability information and the motivations behind it across different populations. Additional insights could tell us, for example, whether traceability policies may impact different consumer groups differently. Our screening criteria expressly included consumers with at least one device with QR-code reading capabilities. As shown by Li and Messer [51], the transaction costs of scanning a QR code to retrieve product information may be higher for consumers who do not own such devices.

Our study specifically examined potential benefits of accessing blockchain-based traceability related to data security and speed, food safety, origin, and organic certifications in a high-risk food. Consumers may benefit from the ability to more quickly trace a food product to its origin and thereof much more quickly identify where a foodborne disease outbreak occurred compared to more traditional food tracing systems. They may also benefit from being able to receive notifications related to food safety and product recalls. Besides food safety, information on the movement of the product through the supply chain may provide value for those consumers who are especially interested in where and by whom the product was grown and what commercial actors played a role in getting the product to market. Our food tracing scenario also allowed QR scanners to view the organic certification (by the USDA) of organic products. However, Shew et al. [20] found that consumers already hold USDA certification by itself in high value independently of blockchain verification. Hence, while the ability to view that certification through QR code scanning may benefit some consumers, it is unclear whether most of the additional value comes from the other benefits of food tracing we highlighted. Future research could dissect the types of information that consumers value and consider other food products for which traceability will become increasingly important.

For supply chain stakeholders, it is worth noting that consumers also valued access to food tracing information through standard (non-blockchain) QR codes relative to not having a QR code, and the costs of providing food tracing information may differ significantly between implementing and maintaining a standard traceability QR code and a QR code fed by a blockchain system. In addition, the successful functioning of farm-to-market traceability systems for different agri-food supply chains, including those based on blockchain technology, may rely on the willingness of supply chain participants to cooperate and share relevant information [52].

We also examine the ongoing U.S. government-industry voluntary provenance labeling in romaine and its impacts on preferences for other leafy greens. We do not find the potential to capture price premiums for leafy greens by labeling the region or sub-region if leafy greens are grown domestically, even though different sub-regions in California and Arizona have been associated with leafy-green foodborne outbreaks. However, if the growing region is labeled, U.S. consumers may discount imported relative to domestically produced leafy greens. Future research on provenance labeling could investigate whether savings to producers and government agencies may still accrue from conforming to voluntary provenance labeling guidelines if a foodborne illness outbreak occurs, their region is not impacted, and their product is not recalled due to their labeling.

Regarding modeling, we find significant heterogeneity in attribute parameters, including in the price parameter, and that the inclusion of an error component improves model fit. While estimating MWTP based on RPL models with uncorrelated random taste parameters might still be the state of practice when modeling food choice behavior, we find that unrestricted RPL models that allow for correlation across random parameters provided the best model fit. Hence, like other researchers in environmental economics [44], we add more evidence to the literature encouraging food choice researchers to also consider the performance of specifications with correlated random parameters.

## Supporting information

S1 File About Blockchain Technology. (PDF)

S2 File About Blockchain in the Food Industry. (PDF)

S3 File Cheap Talk Script. (PDF)

S4 File Econometric Model Sensitivity Analysis. (PDF)

S5 File Econometric Model Heterogeneity Analysis. (PDF)

S6 File Example Choice Set for Romaine Lettuce. (PDF)

S7 File Example Choice Set for Spinach. (PDF)
