# Supplementary material for: Consumer willingness-to-pay for blockchain-based QR code traceability of leafy greens
Source: PLoS One. 2025 Oct 8;20(10):e0331614. doi: 10.1371/journal.pone.0331614 (PMC12507238; doi:10.1371/journal.pone.0331614)
Supplement: S1 File — (PDF) [file pone.0331614.s001.pdf]

S1 File. About Blockchain Technology

Let's learn about **blockchain technology** and its use in the **food industry**.

Blockchain technology is an **electronic record-keeping system** designed to prevent the changing of any digital information recorded in it.

A group of people (or companies) can form a network, record their digital transactions in a blockchain system, and **share** access to it. If a network participant adds information to the record, other participants are notified and must **agree** on whether the new information is accurate before all copies of the record get updated. Information is **encrypted** so that only those authorized can read it. Also, information cannot be changed once added. If a participant adds wrong information, new information documenting the error can be added, but the error and its correction will both be visible to other participants. In theory, these features make blockchain systems a more secure way to manage digital information than standard (non-blockchain) systems.

You may have heard of blockchain in relation to cryptocurrencies like Bitcoin, but blockchain and Bitcoin aren't the same. Blockchain is the electronic record-keeping system used by Bitcoin, but blockchain is used in other contexts as well.

Once you're done reviewing, please proceed.
