# Supplementary material for: Consumer willingness-to-pay for blockchain-based QR code traceability of leafy greens
Source: PLoS One. 2025 Oct 8;20(10):e0331614. doi: 10.1371/journal.pone.0331614 (PMC12507238; doi:10.1371/journal.pone.0331614)
Supplement: S2 File — (PDF) [file pone.0331614.s002.pdf]

S2 File. About Blockchain in the Food Industry

One relatively new use of blockchain is in the **tracing of food products**. That is, food companies can use a blockchain system to record the movement of a food product through the various steps in its supply chain. For example, a group of growers, processors, shippers, and retailers could upload information (records or transactions) about a product and its ingredients into a blockchain traceability system at every step of the product's journey from farm to store (see illustration).

While food companies can opt to use **standard (non-blockchain) systems** for food tracing, companies can also use **blockchain systems**, which means that the information cannot be manipulated once recorded. And because a blockchain system is shared, participants might be able to more quickly trace a food product to its origin, which can help identify, for example, where a foodborne disease outbreak occurred.

Regardless of the system used to record the information, companies could share food tracing and other information with consumers using **QR codes**.

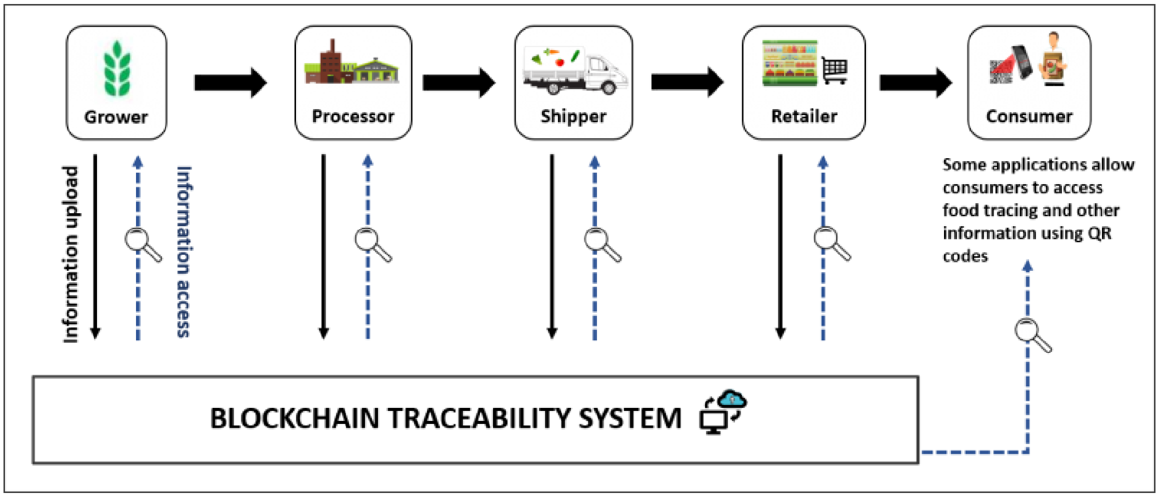

Once you're done reviewing, please proceed.
