# Supplementary material for: Consumer willingness-to-pay for blockchain-based QR code traceability of leafy greens
Source: PLoS One. 2025 Oct 8;20(10):e0331614. doi: 10.1371/journal.pone.0331614 (PMC12507238; doi:10.1371/journal.pone.0331614)
Supplement: S3 File — (PDF) [file pone.0331614.s003.pdf]

S3 File. Cheap Talk Script

Thank you for your participation so far! Next, we're going to ask you a series of choice questions, each of which displays 3 products. For each question, you'll be able to choose which of the 3 products, if any, you're most likely to buy given the prices and information presented for each. However, before you proceed, please read on.

Past studies show that people often state a higher willingness to pay for a product when asked in a survey than what they're actually willing to pay for the product in the store. For instance, a study asked people whether they would hypothetically purchase a new food product at a given price. A large percentage of people said they would buy the new product, but when in reality a grocery store stocked it, only about half of the people bought it. This difference in the way people respond to hypothetical purchase questions as compared to real situations is known as **hypothetical bias**.

So, it is very important that today you **make each of your upcoming choices as if you were really choosing among these products in a grocery store, and actually had to pay the given price to take the product home.**

When you buy a product in the store, you then have less money to spend on other things, so when making each of your choices, please don't forget that there are other things you could spend your money on instead.

Once you're done reviewing, please proceed.
