# Supplementary material for: Consumer willingness-to-pay for blockchain-based QR code traceability of leafy greens
Source: PLoS One. 2025 Oct 8;20(10):e0331614. doi: 10.1371/journal.pone.0331614 (PMC12507238; doi:10.1371/journal.pone.0331614)
Supplement: S4 File — (PDF) [file pone.0331614.s004.pdf]

S4 File. Econometric Model Sensitivity Analysis

We conduct sensitivity testing using a conditional logit model (CL) and a variety of random parameter logit (RPL) model specifications for panel data. Model 1 is a basic CL model, and Model 2 is a RPL model with independent random parameters and a fixed price parameter, both benchmark specifications widely used in DCE studies. Model 3 is identical to Model 2 except that it allows for preference heterogeneity in the price parameter by assuming it is lognormally distributed. Models 4, 5, and 6 introduce non-zero correlations across random parameters. Model 4 is a RPL model with correlated random parameters and a fixed price parameter, and Model 5 is a RPL model with correlated random parameters and a lognormally distributed price parameter. Model 6 is identical to Model 5 except that it allows for correlation across the utilities of the buying options by including an error component.

For each choice experiment, the utility that respondent *i* gets from choosing option *j* within choice set *t* can generally be specified as:

$$U_{ijt} = \beta_{ASC}NoBuy_{ijt} + \beta_p Price_{ijt} + \beta_{1,i}Organic_{ijt} + \beta_{2,i}BlockchainQR_{ijt} + \beta_{3,i}NoQR_{ijt} + \beta_{4,i}CA\_Known_{ijt} + \beta_{5,i}AZ\_Known_{ijt} + \beta_{6,i}AZ\_Unknown_{ijt} + \beta_{7,i}MX\_Known_{ijt} + \beta_{8,i}MX\_Unknown_{ijt} + \eta_{it} + e_{ijt}$$

with variables and parameters as defined in the main body of the paper.

Using a conditional logit framework and modeling all β parameters as fixed results in Model 1. Using a RPL framework and modeling $\beta_1 - \beta_8$ as independent normally distributed random parameters results in Model 2. Using a RPL framework and modeling $\beta_1 - \beta_8$ as normally distributed random parameters, β_p as a lognormally distributed random parameter and all random parameters as independent results in Model 3. Allowing for non-zero correlation among the random parameters in Model 2 results in Model 4 and allowing for non-zero correlation among the random parameters in Model 3 results in Model 5. Lastly, adding an error component (η_{it}) to what would otherwise be Model 5 results in Model 6.

Table A shows the regression results for the romaine lettuce sample. The standard deviation of the price parameter is highly significant in each model in which this parameter is specified to be random (models 3, 5, and 6). There is also strong evidence that the non-price attribute parameters are random. All attribute parameters had statistically significant standard deviations in each model with correlated random parameters (models 4, 5, and 6), and only the California and Arizona sub-region parameters did not have significant standard deviations in the models with uncorrelated random parameters (models 2 and 3). Aligned with the literature, the model with all parameters specified as random and including an error component for the purchase options (model 6) provides the best model fit by all measures (bottom of the table). As for the estimates of the parameters (or their means) in the top half of the table, there is a strong consistency of which parameters are significant across the models. Notably, the price parameter (or its mean) is highly significant and negative in all models (though recall it is restricted to be negative in models in which it is random), providing strong evidence of price validity (a higher price decreasing utility, all else equal) in the data. The *No-buy* constant is negative and significant, indicating a tendency for respondents to choose to purchase a package of romaine lettuce, all else equal.

From the attribute parameter estimates, we conclude that respondents prefer organic to non-organic lettuce, though the standard deviations on organic are large relative to their means, indicating considerable preference heterogeneity. For the QR code attributes, respondents prefer a QR code with traceability information verified with blockchain technology to the (omitted) standard QR code with non-blockchain-verified information. However, they prefer some QR code to none at all. Notably, there is preference heterogeneity in these QR code preferences. Regarding provenance labeling, recall that we use California with unspecified sub-regions as the omitted reference level. The parameters on the California and Arizona regions are generally not significant, suggesting no strong preference for sub-regions (in California) or regions (California vs. Arizona) of origin that are within the United States. However, the parameters on Mexico are negative and significant (again, with wide preference heterogeneity as indicated by the standard deviations when the parameters are specified to be random). Hence, while voluntary provenance labeling may not result in consumers strongly preferring leafy greens grown in one state over another, provenance labeling may result in U.S. consumers discounting imported products relative to domestically produced leafy greens.

Table B displays the regression results for the spinach sample. The conclusions we draw from the spinach results essentially mirror those for romaine lettuce. Again, there is strong evidence of preference heterogeneity for the price parameter and the various product attribute parameters, with respondents preferring organic spinach with blockchain-based QR code tracking, grown in the United States. Model 6, with correlated random parameters and an error component fits best. Because it fits best for both products, we focus our attention in the main paper on Model 6.

Table A. Model Estimation Results for Romaine Choice Experiment

	**Model 1** CL	**Model 2** RPL Independent	**Model 3** RPL Independent Price LN	**Model 4** RPL Correlated	**Model 5** RPL Correlated Price LN	**Model 6** RPL+EC Correlated Price LN
	Parameter (Clust. SE)	Parameter (Clust. SE)	Parameter (Clust. SE)	Parameter (Clust. SE)	Parameter (Clust. SE)	Parameter (Clust. SE)
**Mean**						
Organic	0.712***	0.827***	0.966***	0.808***	0.896***	0.921***
	0.050	0.083	0.092	0.092	0.109	0.106
Blockchain QR code	0.310***	0.249***	0.315***	0.200***	0.253***	0.293***
	0.044	0.064	0.070	0.070	0.085	0.086
No QR code	-0.870***	-1.181***	-1.267***	-1.178***	-1.207***	-1.212***
	0.054	0.082	0.089	0.085	0.094	0.097
CA sub-region known	0.069	0.060	0.047	0.134	0.089	0.058
	0.059	0.078	0.085	0.092	0.106	0.107
AZ sub-region known	0.049	0.120	0.113	0.164*	0.167	0.187*
	0.055	0.076	0.083	0.091	0.112	0.112
AZ sub-region unknown	-0.090	-0.047	-0.059	0.005	0.013	0.069
	0.064	0.081	0.083	0.100	0.116	0.118
MX sub-region known	-0.824***	-0.864***	-0.980***	-0.704***	-0.846***	-0.812***
	0.076	0.098	0.099	0.123	0.157	0.154
MX sub-region unknown	-0.867***	-0.979***	-1.178***	-0.918***	-1.072***	-1.034***
	0.082	0.110	0.120	0.136	0.171	0.166
Price	-0.631***	-0.682***	-0.318***	-0.681***	-0.324***	-0.355***
	0.038	0.038	0.056	0.041	0.064	0.072
No-Buy	-3.557***	-3.750***	-4.963***	-3.767***	-4.934***	-6.346***
	0.169	0.158	0.158	0.172	0.194	0.389
SD of Error Component (EC)						2.793***
						0.352
**SD of random parameters[a]**						
Organic		1.394***	1.305***	1.605***	1.491***	1.434***
		0.083	0.089	0.092	0.128	0.206
Blockchain QR code		0.877***	0.732***	0.907***	0.730***	0.765***
		0.073	0.065	0.081	0.116	0.153
No QR code		1.065***	1.112***	1.016***	1.059***	0.991***
		0.089	0.092	0.096	0.248	0.230
CA sub-region known		0.077	0.080	0.755***	0.683**	0.636**
		0.409	0.745	0.145	0.274	0.312
AZ sub-region known		0.032	0.008	0.806***	0.875***	0.847***
		0.230	1.096	0.205	0.208	0.223

	Model 1<br>CL	Model 2<br>RPL<br>Independent	Model 3<br>RPL<br>Independent<br>Price LN	Model 4<br>RPL<br>Correlated	Model 5<br>RPL<br>Correlated<br>Price LN	Model 6<br>RPL+EC<br>Correlated<br>Price LN
AZ sub-region unknown		0.269	0.088	0.998***	0.966***	0.920***
		0.222	0.665	0.192	0.185	0.271
MX sub-region known		0.665***	0.631***	1.599***	1.749**	1.589**
		0.125	0.148	0.218	0.783	0.740
MX sub-region unknown		1.093***	1.204***	1.863***	2.097**	1.908**
		0.127	0.133	0.304	0.881	0.794
Price			0.548***		0.609***	0.714***
			0.046		0.060	0.082
No. of observed choices (N)	3,968	3,968	3,968	3,968	3,968	3,968
Log-Likelihood	-4,798.07	-4,498.99	-4,370.48	-4,366.48	-4,218.55	-4,178.05
AIC	9,616.10	9,034.00	8,779.00	8,825.00	8,547.10	8,468.10
BIC	9,679.00	9,147.10	8,898.40	9,114.10	8,892.80	8,820.10

[a]All random parameters normally distributed, except for price which is lognormally (LN) distributed. Estimated random parameters in all RPL models are based on 2,000 Halton draws.

***, **, * indicate statistical significance at the 1%, 5%, and 10% levels, respectively.

Table B. Model Estimation Results for Spinach Choice Experiment

	Model 1<br>CL	Model 2<br>RPL<br>Independent	Model 3<br>RPL<br>Independent<br>Price LN	Model 4<br>RPL<br>Correlated	Model 5<br>RPL<br>Correlated<br>Price LN	Model 6<br>RPL+EC<br>Correlated<br>Price LN
	Parameter<br>(Clust. SE)	Parameter<br>(Clust. SE)	Parameter<br>(Clust. SE)	Parameter<br>(Clust. SE)	Parameter<br>(Clust. SE)	Parameter<br>(Clust. SE)
**Mean**						
Organic	0.819***	1.102***	1.151***	1.150***	1.208***	1.169***
	0.056	0.087	0.089	0.094	0.107	0.125
Blockchain QR code	0.303***	0.315***	0.363***	0.329***	0.377***	0.358***
	0.041	0.067	0.063	0.072	0.089	0.095
No QR code	-0.746***	-1.042***	-1.013***	-1.092***	-1.082***	-1.074***
	0.056	0.078	0.078	0.082	0.096	0.105
CA sub-region known	0.031	0.072	0.070	0.187*	0.101	0.114
	0.059	0.086	0.092	0.098	0.124	0.140
AZ sub-region known	0.054	0.100	0.084	0.213**	0.222*	0.287**
	0.068	0.082	0.082	0.101	0.123	0.135
AZ sub-region unknown	-0.097	-0.096	-0.104	-0.074	-0.046	0.027
	0.073	0.081	0.084	0.107	0.119	0.135
MX sub-region known	-0.747***	-0.958***	-0.978***	-0.972***	-1.013***	-1.042***
	0.086	0.102	0.101	0.126	0.146	0.165

	Model 1 CL	Model 2 RPL Independent	Model 3 RPL Independent Price LN	Model 4 RPL Correlated	Model 5 RPL Correlated Price LN	Model 6 RPL+EC Correlated Price LN
MX sub-region unknown	-0.810***	-1.254***	-1.215***	-1.200***	-1.208***	-1.255***
	0.078	0.116	0.118	0.130	0.149	0.166
Price	-0.515***	-0.679***	-0.469***	-0.743***	-0.341***	-0.605***
	0.035	0.032	0.053	0.034	0.053	0.100
No-Buy	-3.283***	-3.867***	-5.445***	-4.328***	-5.632***	-10.616***
	0.166	0.137	0.125	0.152	0.164	0.676
SD of Error Component (EC)						5.930***
						0.639
**SD of random parameters[a]**						
Organic		1.433***	1.312***	1.575***	1.449***	1.512***
		0.083	0.090	0.093	0.112	0.126
Blockchain QR code		0.842***	0.526***	0.890***	0.657***	0.702***
		0.076	0.078	0.083	0.096	0.096
No QR code		0.998***	1.007***	1.050***	1.096***	1.025***
		0.089	0.099	0.090	0.107	0.124
CA sub-region known		0.523***	0.325	0.978***	0.776***	0.703***
		0.149	0.210	0.175	0.181	0.220
AZ sub-region known		0.288	0.166	1.154***	1.149***	0.909***
		0.228	0.383	0.175	0.174	0.184
AZ sub-region unknown		0.034	0.007	1.317***	1.202***	0.881***
		0.325	1.235	0.194	0.170	0.188
MX sub-region known		0.820***	0.787***	1.654***	1.742***	1.663***
		0.138	0.150	0.168	0.177	0.195
MX sub-region unknown		1.301***	1.192***	1.735***	1.663***	1.721***
		0.130	0.134	0.143	0.193	0.248
Price			0.715***		0.704***	1.317***
			0.054		0.061	0.099
No. of observed choices (N)	3,984	3,984	3,984	3,984	3,984	3,984
Log-Likelihood	-4,671.09	-4,385.77	-4,150.77	-4,226.48	-4,043.11	-3,944.92
AIC	9,362.20	8,807.50	8,339.50	8,545.00	8,196.20	8,001.80
BIC	9,425.10	8,920.80	8,459.00	8,834.30	8,542.20	8,354.10

[a]All random parameters normally distributed, except for price which is lognormally (LN) distributed. Estimated random parameters in all RPL models are based on 2,000 Halton draws.

***, **, * indicate statistical significance at the 1%, 5%, and 10% levels, respectively.
