# Supplementary material for: Consumer willingness-to-pay for blockchain-based QR code traceability of leafy greens
Source: PLoS One. 2025 Oct 8;20(10):e0331614. doi: 10.1371/journal.pone.0331614 (PMC12507238; doi:10.1371/journal.pone.0331614)
Supplement: S5 File — (PDF) [file pone.0331614.s005.pdf]

S5 File. Heterogeneity Analysis Results

To obtain some insight into what the subset of the population with a positive willingness to pay for blockchain-based QR codes on food packaging looks like, we used the "*;par*" and "*;WTP*" commands in NLOGIT 6 to calculate an individual-specific marginal willingness to pay value for each respondent in our survey sample, for each product.

Table C. Logit Regression Results, Dependent Variables is Individual-Specific MWTP for Blockchain-Based QR Code

Variable	Definition	Romaine sample			Spinach sample		
		Estimate	SE		Estimate	SE	
Age	Age, measured in years	0.01	0.01		0.02	0.01	
Female	1 if female; 0 otherwise	-0.04	0.23		0.16	0.29	
Black race identity	1 if identifies as Black or African American only; 0 otherwise	0.50	0.32		-0.41	0.32	
Another or multiple race identity	1 if identifies as another race or multiple races; 0 otherwise	-0.74	0.51		0.15	0.71	
Bachelor's	1 if highest educational attainment is bachelor's degree; 0 otherwise	-0.07	0.29		-0.29	0.31	
Associate	1 if highest educational attainment is 2-year or associate degree; 0 otherwise	-0.11	0.33		0.18	0.40	
Household size	No. of persons per household	0.02	0.12		0.34	0.18	*
Has children	1 if has children 17 or younger living in household; 0 otherwise	0.06	0.34		-1.32	0.49	***
Income	Household income, measured in $1,000s	-0.01	0.00	**	-0.01	0.00	
Part- or full-time employed	1 if employment status is part- or full-time employed; 0 otherwise	0.31	0.38		0.11	0.54	
Stay at home parent or retired	1 if employment status is stay at home parent or retired; 0 otherwise	0.12	0.44		-0.46	0.58	
Married	1 if married; 0 otherwise	0.75	0.29	***	-0.28	0.38	
South	1 if ZIP code is in the South; 0 otherwise	-0.45	0.27	*	-0.28	0.31	
West	1 if ZIP code is in the West; 0 otherwise	-0.14	0.32		0.20	0.42	
Midwest	1 if ZIP code is in the Midwest; 0 otherwise	-0.61	0.32	*	-0.39	0.41	
Prior blockchain knowledge	1 if prior blockchain awareness & ≥50% correct on pre-information quiz; 0 otherwise	-0.19	0.27		-0.11	0.31	
Prior QR code knowledge	1 if correctly identified a QR code image; 0 otherwise	-0.13	0.29		0.96	0.32	***
Frequency of QR code scanning in-store	No. of times QR codes on food packaging were scanned in-store in past year	0.02	0.03		0.00	0.03	
Frequency of QR code scanning at home	No. of times QR codes on food packaging were scanned at home in past year	0.05	0.03		0.06	0.03	*
Food safety concerns	1 if concerned about improving the safety of the U.S. food supply; 0 otherwise	1.22	0.36	***	0.26	0.45	

Food origin interest	1 if interested in how food systems work and wants to know food origin; 0 otherwise	-0.02	0.35	-0.50	0.49
Frequent purchaser of non-organic romaine/spinach	1 if buys at least 1 package of corresponding non-organic product ≥ thrice/month; 0 otherwise	-0.10	0.25	-0.19	0.30
Frequent purchaser of organic romaine/spinach	1 if buys at least 1 package of corresponding organic product≥ thrice/month; 0 otherwise	-0.41	0.27	-0.30	0.29
Constant	Constant term	-0.34	0.88	0.58	1.15
No. of respondents		496		498	
Log-Likelihood		-289.12		-221.40	
LR $\chi^2(23)$		39.44**		45.46***	

***, **, * indicate statistical significance at the 1%, 5%, and 10% levels, respectively.
