# Supplementary material for: Consumer willingness-to-pay for blockchain-based QR code traceability of leafy greens
Source: PLoS One. 2025 Oct 8;20(10):e0331614. doi: 10.1371/journal.pone.0331614 (PMC12507238; doi:10.1371/journal.pone.0331614)

**S7 File. Example Choice Set of Packaged Spinach**

Carefully consider each of the following options for spinach (1 lb. package). Suppose these options were the only ones available in the store. Please choose, by selecting the corresponding image, which of the products you are most likely to buy given the prices and information presented for each:

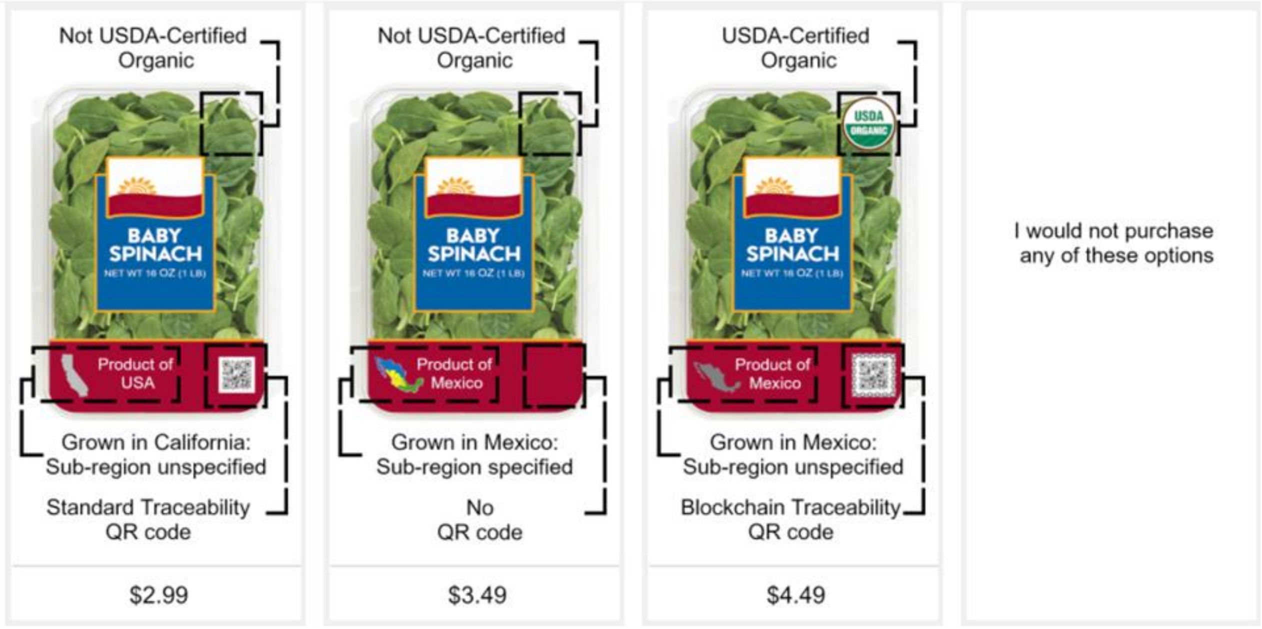

Supplement: S7 File — (PDF) [file pone.0331614.s007.pdf]
